# Supplementary material for: Her2 Expression in Circulating Tumor Cells Is Associated with Poor Outcomes in Patients with Metastatic Castration-Resistant Prostate Cancer
Source: Cancers (Basel). 2021 Nov 29;13(23):6014. doi: 10.3390/cancers13236014 (PMC8656805; doi:10.3390/cancers13236014)
Supplement: Supplementary file 1 [file cancers-13-06014-s001.zip › cancers-1442402-supplementary.pdf]

Supplementary Materials: Her2 Expression in Circulating Tumor Cells Is Associated with Poor Outcomes in Patients with Metastatic Castration-Resistant Prostate Cancer

Denis Maillet, Nathalie Allioli, Julien Péron, Adriana Plesa, Myriam Decaussin-Petrucci, Sophie Tartas, Christophe Sajous, Alain Ruffion, Sébastien Crouzet, Gilles Freyer and Virginie Vlaeminck-Guillem

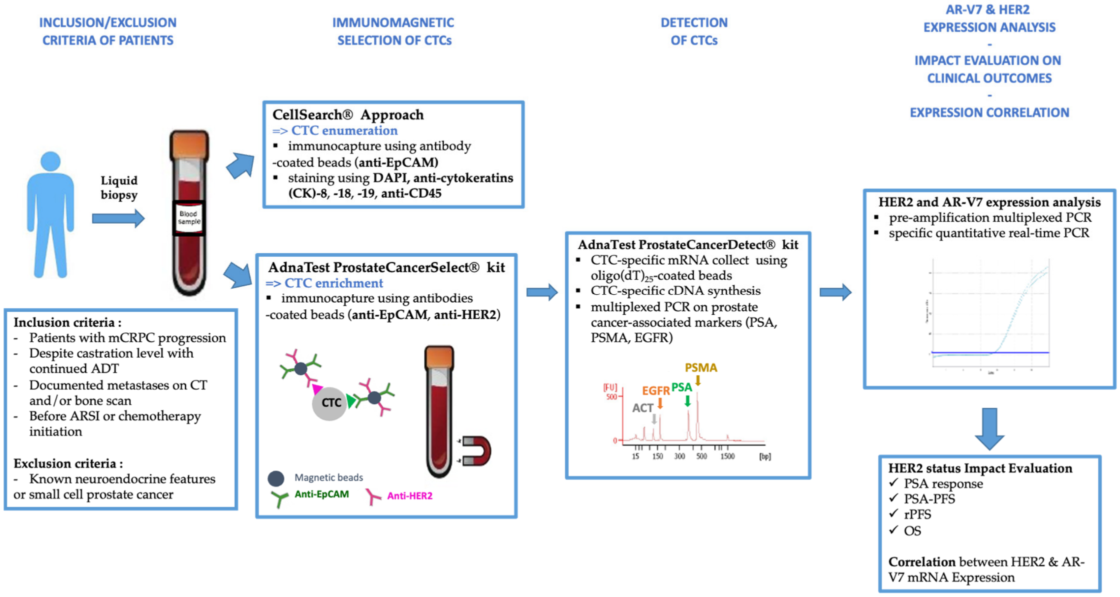

Figure S1. Flow diagram of the experimental approach for Circulating Tumor Cells (CTCs) isolation, detection and CTC-based HER2/AR-V7 expression analysis.

Patients Samples. Whole blood was collected from patients with metastatic castration resistant prostate cancer (mCRPC), regarding inclusion/exclusion criteria (ADT, androgen deprivation therapy; CT, computed tomography; ARSI, androgen receptor signaling inhibitors).
CellSearch® Approach. Circulating tumor cells (CTCs) were immunomagnetically enriched from 7.5 mL of blood through immunocapture and magnetically isolation using Ferrofluid coupled to monoclonal antibody specific for the epithelial cell adhesion molecule (EpCAM), and enumerated under fluorescence microscope analysis after staining with Phycoerthrin-5(PE)-conjugated monoclonal antibodies directed against cytokeratins (CK)-8, 18, 19, allophycocyanin (APC)-conjugated monoclonal antibody identifying CD45 expressed in leukocytes and the nucleic acid dye DAPI (4′, 6-diamidino-2-phenylindole dihydrochloride). The cells exhibit morphological features consistent with of a tumor cell and the phenotype EpCAM+, CK+, DAPI+, CD45- are defined as CTCs in the CellSearch® system.
AdnaTest® Approach. In parallel, CTCs were immunomagnetically enriched from 5 mL of blood through immunocapture and magnetically isolation using magnetic beads coated with a combination of antibodies against epithelial (EpCAM) and tumor-associated (HER2) antigens. After cell lysis, Messenger RNA (mRNA) were magnetically extracted from the cell lysate with oligo(dT)25-coated magnetic beads and fully retrotranscribed to obtain CTC-specific complementary DNA (cDNA). CTC detection was defined on expression analysis of three prostate cancer-associated transcripts (PSA, PSMA, and EGFR genes) performed by a multiplexed PCR reaction using template CTC-specific cDNA. The presence of CTCs was noted if at least one peak related to the three PCR amplicons was detected by microcapillary electrophoresis (Bioanalyzer, Agilent, Santa Clara, California, USA).
Expression analysis and impact evaluation on clinical outcomes. Expression analysis of HER2 and AR-V7 was performed using template CTC-specific cDNA, by pre-amplification multiplex PCR (allowing simultaneous targeted genes amplification), followed by specific quantitative real-time PCR reactions using the pre-amplified PCR samples. Impact of HER status on clinical outcomes was evaluated.

Table S1. – PSA-PFS prognostic factors among patients treated with androgen receptor signaling inhibitors (ARSI) in univariate and multivariate analysis (Cox model).

**Cox Proportional Hazards Regression for PSA-PFS**					
		**Unadjusted Analysis**		**Adjusted Analysis**	
**Circulating Biomarker**	**N (%)**	**HR (95% CI)**	***p*-value**	**HR (95% CI)**	***p*-value**
AR-V7+	27/31 (87%)	2.02 (0.9–4.4)	0.075	2.22 (0.98–7.8)	0.06
Detection of CTCs (AdnaTest®)	27/31 (87%)	3.88 (1.4–10.8)	0.008	2.74 (0.98–5.0)	0.06
HER2+	27/31 (87%)	3.35 (1.3–8.7)	0.013	2.87 (1.01–8.1)	0.047

Table S2. Radiological-PFS (rPFS) prognostic factors among patients treated with androgen receptor signaling inhibitors (ARSI) in univariate and multivariate analysis (Cox model).

**Cox Proportional Hazards Regression for rPFS**					
		Unadjusted Analysis		Adjusted Analysis	
Circulating Biomarker	N (%)	HR (95% CI)	*p*-value	HR (95% CI)	*p*-value
AR-V7+	23/31 (74%)	4.26 (1.4–12.9)	0.01	3.22 (1.3–7.8)	0.01
Detection of CTCs (AdnaTest®)	23/31 (74%)	3.25 (1.1–9.6)	0.033	2.69 (0.9–8.3)	0.08
HER2+	23/31 (74%)	3.35 (1.3–8.7)	0.013	3.62 (1.1–11.6)	0.03
